# Case report end-stage cystic fibrosis with fatal outcome in two siblings from a Chinese family: a 15-year diagnostic odyssey

**DOI:** 10.3389/fphar.2026.1894322

**Published:** 2026-09-08

**Authors:** Yuelin Shen, Qionghua Chen, Haiming Yang

**Affiliations:** 1 Respiratory Department, National Clinical Research Center for Respiratory Diseases, Beijing Children’s Hospital, Capital Medical University, National Center for Children’s Health, Beijing, China; 2 Respiratory Department, Quanzhou Maternal and Child Health Hospital (Quanzhou Children’s Hospital), Quanzhou, China

**Keywords:** CFTR, CFTR modulators, China, cystic fibrosis, diagnostic delay, east asian-specific CFTR variants, theratyping

## Abstract

**Background:**

Cystic fibrosis (CF) remains severely underdiagnosed in China due to the absence of newborn screening, limited sweat chloride testing capacity, and low clinical awareness. Diagnostic delay results in irreversible lung damage and precludes timely genotype-directed therapy.

**Case Presentation:**

A 16-year-old Chinese boy presented with end-stage CF lung disease after a 15-year diagnostic odyssey. He had recurrent lower respiratory tract infections since infancy, progressive bronchiectasis, CF-associated liver disease with portal hypertension, and chronic *Pseudomonas aeruginosa*/Burkholderia cepacia co-infection. Sweat chloride was 102 mmol/L. Whole-exome sequencing identified compound heterozygous CFTR variants: c.579 + 1_579+2insACAT (a canonical splice-site insertion, predicted null) inherited from the father, and c.1766 + 5G>T (CF-causing in CFTR2, near-complete exon 13 skipping) inherited from the mother. Fecal elastase was preserved (525 μg/g), confirming pancreatic sufficiency; this finding is consistent with but does not prove residual CFTR function. CFTR modulator therapy was unavailable. He died of cardiopulmonary failure approximately 1 year after diagnosis while awaiting lung transplantation. Cascade testing confirmed the same genotype in his 11-year-old sister (sweat chloride 111 mmol/L), who also died of cardiopulmonary failure within months. Their eldest brother had died at age two of severe pneumonia without CF being suspected.

**Conclusion:**

This family illustrates the convergence of three pharmacologically relevant barriers in underserved CF populations: prolonged diagnostic delay precluding specialized care, uncharacterized population-specific variants lacking theratyping data, and inaccessibility of CFTR modulators. The preserved pancreatic function despite end-stage lung disease is compatible with residual CFTR activity, but the contribution of either allele and potential responsiveness to CFTR modulators require transcript, protein, and functional characterization. This underscores the urgent need for systematic theratyping of under-characterized CFTR variants reported in East Asian populations. Expansion of sweat chloride testing, clinician education, and international collaborative theratyping efforts are needed to break this cycle.

## 1 Introduction

Cystic fibrosis (CF) remains severely underdiagnosed in populations without newborn screening. In China, CF newborn screening has not been implemented, and sweat chloride testing, the diagnostic gold standard (Farrell et al., 2017), is available at fewer than five centers nationwide. Consequently, only 202 genetically confirmed Chinese CF patients were reported between 1993 and 2022, based on a comprehensive literature review and our center’s registry (Shen et al., 2022), whereas prevalence modeling estimates the true number exceeds 20,000 (Chinese Experts Cystic Fibrosis Consensus Committee; Chinese Alliance for Rare Lung Diseases; Chinese Alliance for Rare Diseases, 2023). This disparity is largely infrastructural: most clinicians outside tertiary referral centers have never encountered a confirmed case, and whole-exome sequencing (WES), often the sole path to diagnosis, costs US $700–1,300, requires 3–5 weeks, and is frequently not reimbursed by public insurance (Chinese Experts Cystic Fibrosis Consensus Committee; Chinese Alliance for Rare Lung Diseases; Chinese Alliance for Rare Diseases, 2023).

Herein, we report a family in which two genetically confirmed siblings died of CF-related respiratory failure after a 15-year diagnostic odyssey. Their eldest brother, who died of severe respiratory disease at age 2, is retrospectively suspected to have had CF but was never tested. The proband, a 16-year-old boy, presented with end-stage CF lung disease, CF-associated liver disease (CFLD), and fatal cardiopulmonary failure. His case, together with the confirmed death of his younger sister from the same genotype and the suspected CF-related death of their eldest brother, underscores the urgent need to expand CF diagnostic and therapeutic infrastructure in countries where the disease remains hidden.

## 2 Case presentation

**FIGURE 1:**
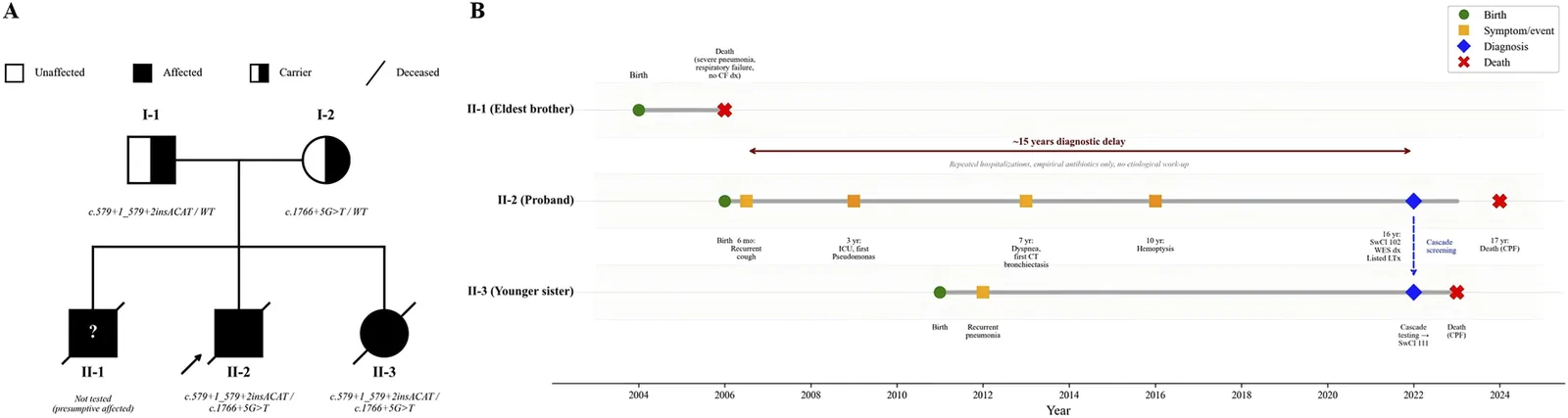
Family pedigree and clinical timeline **(A)** Pedigree of the affected family. Filled symbols indicate affected individuals; half-filled symbols indicate obligate heterozygous carriers confirmed by Sanger sequencing. The question mark denotes presumptive affected status (no genetic testing performed). A diagonal line indicates deceased individuals. The arrow indicates the proband (II-2). Genotypes are shown below each individual. The parents are non-consanguineous **(B)** Clinical timeline of two genetically confirmed siblings and one presumptively affected sibling plotted on a shared calendar-year axis. Green circles denote birth; orange squares denote clinical symptoms or events; blue diamonds denote diagnosis; red X markers denote death. The red double-headed arrow indicates the ∼15-year interval from symptom onset to diagnosis in the proband, during which he received repeated hospitalizations with empirical antibiotics only and no etiological work-up. The blue dashed arrow indicates cascade genetic screening from the proband to his younger sister. CPF, cardiopulmonary failure; SwCl, sweat chloride; WES, whole-exome sequencing; LTx, lung transplantation.

A 16-year-old boy was referred to our center with progressive respiratory insufficiency. He had a history of recurrent lower respiratory tract infections since infancy and had required intensive care for pneumonia at age 3. Exercise intolerance and exertional dyspnea worsened progressively from age 7, and hemoptysis occurred at age 10. Growth had been persistently poor throughout childhood. The parents are non-consanguineous. Family history included an older brother who had died of severe pneumonia with respiratory failure at 2 years of age (undiagnosed, no genetic testing performed) and a younger sister with chronic productive cough and recurrent pneumonia (Figure 1A).

On admission, he was tachypneic (respiratory rate, 36/min) with oxygen saturation of 92% on 5 L/min supplemental oxygen. Height was 150 cm and weight was 38 kg (both below the 3rd percentile; BMI 16.9 kg/m^2^). Physical examination revealed marked digital clubbing and bilateral diminished breath sounds.

**FIGURE 2:**
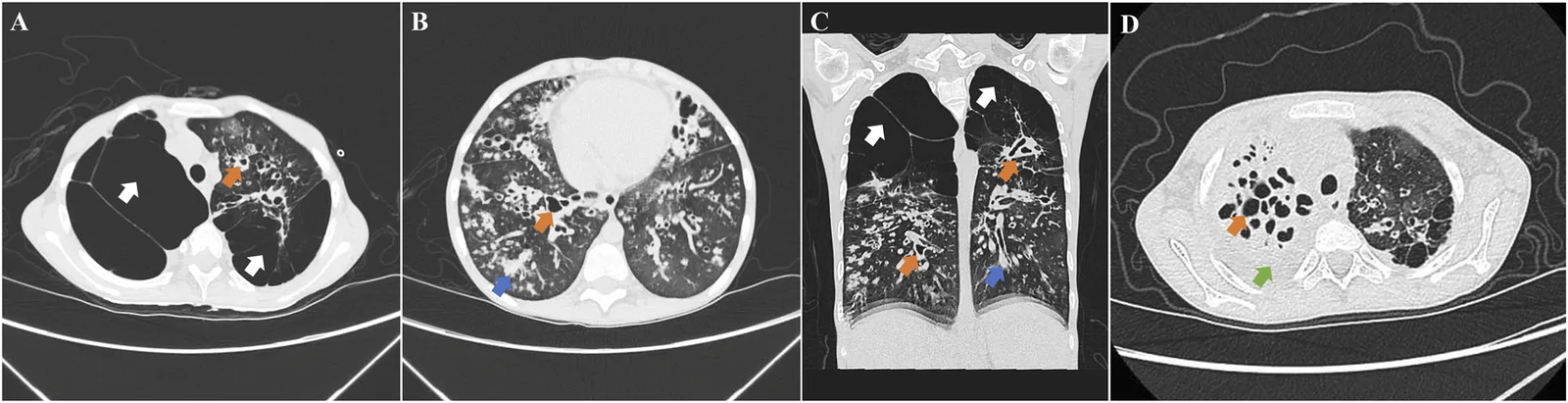
Chest computed tomography of two siblings with end-stage cystic fibrosis. White arrows indicate giant bullae; orange arrows indicate bronchiectasis; blue arrows indicate mucus plugging; green arrows indicate consolidation **(A)** Proband (16-year-old boy): axial image at the level of the upper lobes showing giant bullae in both upper lobes with near-complete parenchymal destruction **(B)** Proband: axial image at the level of the lower lobes demonstrating bilateral diffuse bronchiectasis, mucus plugging **(C)** Proband: coronal reconstruction showing the full craniocaudal extent of lung destruction, with upper-lobe bullous changes and lower-lobe bronchiectasis **(D)** Younger sister (11-year-old girl): axial image showing extensive consolidation of the right upper lobe with cystic bronchiectasis within the consolidated parenchyma.

Pulmonary function testing demonstrated moderate restriction with severe obstruction: FEV_1_ 28.2% predicted, FVC 43.9% predicted, FEV_1_/FVC 65.2%, PEF 31.3% predicted, and FEF_25-75_ 10.2% predicted. Arterial blood gas analysis demonstrated severe hypercapnia (PaCO_2_ 92.9 mmHg; normal, 35–45 mmHg). Chest CT revealed extensive bilateral bronchiectasis with mucus plugging and large bullous changes in the upper lobes (Figures 2A–C). Sputum cultures grew *Pseudomonas aeruginosa*, *Burkholderia cepacia complex*, and *Candida albicans*. The *Burkholderia* isolate was further identified as *B. cepacia* by metagenomic next-generation sequencing. Serum (1,3)-β-D-glucan was markedly elevated (898 pg/mL; normal, <60 pg/mL), although these findings may reflect airway colonization rather than invasive fungal disease.

Laboratory evaluation revealed mildly elevated transaminases (ALT 52.8 U/L, AST 60.6 U/L) with elevated cholestatic markers (GGT 106.6 U/L, ALP 370 U/L). Serum albumin was 24.6 g/L (normal 35–50 g/L), attributed primarily to CF-related chronic malnutrition and systemic inflammation; hepatic synthetic function was otherwise preserved (INR 1.06, total bilirubin 5.6 μmol/L, platelet count 162 × 10^9^/L; calculated MELD score 7). Abdominal ultrasound revealed heterogeneous hepatic echotexture with portal vein dilatation and splenomegaly, consistent with CFLD and portal hypertension. Echocardiography demonstrated moderate pulmonary hypertension (tricuspid regurgitation pressure gradient 45 mmHg). Fecal elastase-1 was 525 μg/g (formed stool specimen; normal >200 μg/g), confirming exocrine pancreatic sufficiency. Sweat chloride testing was performed using the Macroduct collection system, yielding a value of 102 mmol/L (diagnostic threshold >60 mmol/L). Allergic bronchopulmonary aspergillosis was excluded by normal serum total IgE of 49.8 IU/mL, Aspergillus-specific IgE 0.13 KUA/L, combined with absence of peripheral eosinophilia. Tuberculosis was excluded by negative tuberculin skin test, negative interferon-gamma release assay, and negative sputum analysis. Alpha-1 antitrypsin deficiency was excluded by normal serum alpha-1 antitrypsin level. Primary ciliary dyskinesia (PCD) was considered unlikely based on nasal nitric oxide of 190 nL/min (Sunvou CA2122, velum closure method; PCD screening threshold <77 nL/min); the value is within the reduced range typical of CF (100–250 nL/min).

Given clinical features highly suggestive of CF—diffuse bronchiectasis, CFLD, chronic *Pseudomonas/Burkholderia* co-infection, and markedly elevated sweat chloride—genetic testing was pursued. WES was performed at MyGenostics Inc (Beijing, China) on the Illumina NovaSeq 6,000 platform (paired-end 150 bp; mean depth >100×; >99% of target regions covered at ≥10× and >98% at ≥20×). Variants were called using BWA/GATK HaplotypeCaller against GRCh37/hg19 and annotated based on CFTR reference transcript NM_000492.4. Variants were filtered by minor allele frequency <0.01 in gnomAD and ChinaMAP, predicted functional impact (splice-site, frameshift, nonsense, or missense predicted deleterious by *in silico* tools), and inheritance pattern consistent with autosomal recessive disease. Read-depth-based CNV analysis was performed; no large CFTR deletions or duplications were detected. Two compound heterozygous variants were identified: c.579 + 1_579+2insACAT (a canonical splice-site insertion in intron 5, first reported in Chinese CF patients by our group (Shen et al., 2022) without detailed phenotypic characterization, not yet listed in CFTR2) and c.1766 + 5G>T (legacy: 1898+5G→T; a non-canonical splice-site variant in intron 13, listed as CF-causing in CFTR2). Both were confirmed by Sanger sequencing (ABI 3730xl), and parental segregation analysis confirmed that c.579 + 1_579+2insACAT was inherited from the father and c.1766 + 5G>T from the mother, demonstrating the two variants reside in trans. The proband’s sister was confirmed to carry the same genotype. According to ACMG/AMP criteria (Richards et al., 2015), c.579 + 1_579+2insACAT is classified as Pathogenic (PVS1 + PM2 + PM3 + PP4), and c.1766 + 5G>T is classified as Pathogenic (PS3 + PM2 + PM3 + PP4; functional studies demonstrated near-complete exon 13 skipping [legacy exon 12] (Zielenski et al., 1995; Sun et al., 2024)). No pathogenic variants were identified in genes associated with PCD or primary immunodeficiency. Combined with the normal nasal nitric oxide, normal immunoglobulins, and normal lymphocyte subsets, the clinical and laboratory findings did not support these alternative diagnoses.

Following genetic confirmation, intensive supportive care was provided, including intravenous antibiotics, antifungal therapy, airway clearance techniques, nutritional optimization, and supplemental oxygen. CFTR modulator therapy (elexacaftor/tezacaftor/ivacaftor) was not available, as neither variant is included in current approved indications and the triple combination is not commercially accessible in China. The patient was listed for lung transplantation following multidisciplinary evaluation. The team acknowledged *B.urkholderia cepacia* colonization (a relative contraindication to lung transplantation) as well as additional surgical risk factors including CFLD with portal hypertension and moderate pulmonary hypertension. Given the rapidly fatal trajectory, listing was pursued as the only potentially life-prolonging intervention. He died of cardiopulmonary failure secondary to acute pulmonary infection approximately 1 year after diagnosis, while awaiting a donor organ (Figure 1B).

Following the proband’s diagnosis, targeted Sanger sequencing was performed on his 11-year-old sister. She was confirmed to carry the same compound heterozygous *CFTR* genotype. Her clinical history was similar to that of her brother: recurrent lower respiratory tract infections since infancy, progressive exercise intolerance, and failure to thrive. Height and weight were both below the 3rd percentile. Sweat chloride was markedly elevated at 111 mmol/L. Echocardiography revealed right atrial and right ventricular enlargement with moderate pulmonary hypertension. Chest CT demonstrated diffuse bilateral bronchial wall thickening and bronchiectasis, volume loss in the right upper lobe with consolidation and large cystic changes (Figure 2D). Pulmonary function testing could not be performed due to her critical respiratory status. Sputum cultures grew *P.seudomonas aeruginosa* and *Aspergillus fumigatus*. Serum total IgE was 519 IU/mL with Aspergillus-specific IgE of 0.51 KUA/L; however, peripheral eosinophils were normal, and diagnostic criteria for ABPA were not met. She was initiated on intensive antimicrobial therapy and airway clearance but progressed to cardiopulmonary failure and died within months of diagnosis.

## 3 Discussion

This case illustrates three critical issues facing CF care in populations where the disease is underrecognized. The 15-year interval from symptom onset to diagnosis in our patient is not an outlier in countries without CF newborn screening. Of note, the eldest sibling’s CF diagnosis remains unconfirmed. He died at age two at a local primary care hospital that lacked both sweat chloride testing and genetic testing capabilities, and CF was not considered at that time. His presumptive diagnosis is based on retrospective clinical inference following the genetic confirmation in his two younger siblings. This case itself exemplifies how limited diagnostic awareness and infrastructure perpetuate missed diagnoses across generations within the same family. Among 103 children with CF diagnosed at our center, the mean age at diagnosis was 7.6 years (SD, 4.4) (Shen et al., 2022), compared with a median of less than 2 months in countries with established newborn screening programs (Cystic Fibrosis Foundation. Cystic Fibrosis Foundation P atient Registry, 2022 Annual Data Report, 2023). Diagnostic delay in CF carries direct prognostic consequences: in a 40-year Italian cohort, 20-year survival among patients with severe early presentation was 85% when diagnosed through newborn screening versus 64% when diagnosed after symptoms (p = 0.007) (Padoan et al., 2018). Similar patterns of delayed or missed diagnosis are reported across low- and middle-income countries where CF screening infrastructure is lacking (Fuhrer et al., 2024). This diagnostic delay in our patient did not reflect an atypical presentation; his phenotype was textbook CF from infancy. Rather, it reflected the extremely low prevalence of CF in East Asian populations, the absence of newborn screening in China, and limited access to sweat chloride testing, resulting in low clinical awareness such that these children are typically labeled as having “recurrent pneumonia” or “bronchiectasis of unknown cause” (Chinese Experts Cystic Fibrosis Consensus Committee; Chinese Alliance for Rare Lung Diseases; Chinese Alliance for Rare Diseases, 2023). Notably, the eldest sibling in this family died of severe pneumonia at 2 years of age without CF being considered, and more than a decade elapsed before the proband was eventually diagnosed, demonstrating that even familial clustering failed to trigger diagnostic suspicion. By the time cascade testing identified the same genotype in the younger sister, her disease had already progressed to an irreversible stage. Her case illustrates that delayed diagnosis in an index patient has consequences extending beyond that individual—siblings who might have benefited from early intervention are identified only when therapeutic options are no longer available. The images in this case (Figure 2) depict the endpoint of this diagnostic odyssey: advanced parenchymal destruction that timely diagnosis and appropriate therapy may have mitigated.

From a technical standpoint, both causative variants in this family are intronic—a canonical +1/+2 splice-site insertion and a +5 non-canonical splice variant. These were captured by WES flanking reads because they lie close to exon boundaries; however, this represents a fortunate circumstance rather than a reliable detection strategy. Most diagnostic laboratories use targeted CFTR panels designed around the European/CFTR2 common-mutation set, or exome capture with limited intronic coverage, which would not reliably detect population-specific intronic alleles such as these. Moreover, truly deep-intronic pathogenic CFTR variants (e.g., c.3874–4522 A>G, identified in Chinese CF patients (Shen et al., 2022); c.3718–2477C>T [legacy 3,849 + 10 kb C>T]) are invisible to both exon-focused panels and standard WES, requiring CFTR full-gene sequencing, whole-genome sequencing, or RNA/transcript analysis for detection. In populations with a distinct CFTR mutation spectrum, full-gene or genome-level sequencing—rather than European-biased exon panels—should be the standard diagnostic approach when clinical suspicion is high. This further reinforces the theratyping message, since characterization of splice variants ultimately depends on transcript-level analysis.

The c.579 + 1_579+2insACAT variant disrupts the canonical donor splice site of *CFTR* exon 5 (+1/+2 position) and is predicted to abolish normal splicing, likely producing no functional CFTR protein from that allele. This prediction is supported by the direct disruption of the invariant GT dinucleotide at the +1/+2 position, which is universally required for donor splice-site recognition; however, direct RNA or protein-level confirmation has not been performed. This variant was first identified in Chinese CF patients at our center (Shen et al., 2022) and remains absent from ClinVar, gnomAD, ChinaMAP, and the CFTR2 database, indicating it is an ultra-rare variant previously identified only in Chinese patients. The present case provides the first detailed clinical characterization of this genotype. The c.1766 + 5G>T variant (legacy: 1898+5G→T) is listed in CFTR2 as CF-causing and is the second most frequent *CFTR* variant among Chinese CF patients, predominantly found in southern China (Shen et al., 2022). Functional studies have demonstrated that this variant causes skipping of exon 13 (legacy exon 12), deleting 29 amino acids from the first nucleotide-binding domain and rendering the protein non-functional. The affected allele almost exclusively produces exon-skipped transcript, with no detectable normal mRNA by conventional RT-PCR (Zielenski et al., 1995; Sun et al., 2024).

Despite this established splicing consequence, c.1766 + 5G>T has never been included in CFTR modulator clinical trials and no theratyping data exist. Other *CFTR* splicing variants (e.g., 2,789 + 5G>A, 3272-26 A>G) have been classified as residual-function mutations and are included in the approved indications for ivacaftor (Clancy et al., 2019); whether c.1766 + 5G>T, which causes near-complete exon skipping, could similarly respond to modulators remains unknown.

The preserved fecal elastase in our patient is noteworthy. Despite end-stage pulmonary disease, exocrine pancreatic function remained intact. Preserved fecal elastase confirms pancreatic sufficiency, which is compatible with but does not establish residual CFTR function. The contribution of each allele and potential responsiveness to CFTR modulators remain to be determined through transcript, protein, and functional characterization. Notably, previous RT-PCR studies of c.1766 + 5G>T reported that a low level of correctly spliced transcript cannot be excluded (Zielenski et al., 1995; Sun et al., 2024), but whether this translates to meaningful CFTR protein expression at the cell surface remains unknown. The profound growth failure in this pancreatic-sufficient patient is therefore more plausibly attributable to the energy cost of end-stage suppurative lung disease, chronic polymicrobial airway infection, CFLD, and chronic hypoxia/hypercapnia, rather than fat malabsorption. The hypoalbuminemia (24.6 g/L) further supports a catabolic/hepatic etiology of growth failure rather than pancreatic insufficiency.

Building on the above evidence of residual CFTR function, formal functional characterization is needed to confirm modulator responsiveness and requires a stepwise approach. First, RNA analysis from nasal brushings or intestinal biopsies is required to define the splicing products generated by both variants. Conventional RT-PCR can characterize aberrant splice products, but quantification of low-abundance residual normally spliced transcript requires digital droplet PCR with splice-junction-specific primers for adequate sensitivity. Second, CFTR protein expression and maturation should be assessed by Western blot (band B/C shift); however, the presence of mature band C glycoform does not establish plasma membrane localization. Surface biotinylation assay would be required to confirm that full-length CFTR reaches the apical membrane. Third, functional testing in patient-derived models—nasal epithelial cultures or intestinal organoid forskolin-induced swelling (FIS) assays—would establish baseline CFTR channel activity and enable theratyping with available modulators (Clancy et al., 2019). Importantly, if c.579 + 1_579+2insACAT produces no residual full-length transcript, the dominant defect is aberrant splicing rather than defective protein folding or gating, and conventional corrector/potentiator combinations would not be expected to provide benefit. Specifically, in the absence of a sufficiently expressed, membrane-localized, and pharmacologically responsive CFTR protein product, currently approved CFTR modulators would not be expected to provide clinical benefit. Investigational splice-modulating antisense oligonucleotides could theoretically be explored to promote correct exon inclusion; however, their feasibility depends on the specific nature of the splice defect and the accessibility of the target pre-mRNA sequence, and cannot be determined before transcript-level characterization. For the c.1766 + 5G>T allele, given the possible retention of low-level correctly spliced transcript (Zielenski et al., 1995; Sun et al., 2024), conventional CFTR modulators could be effective if sufficient full-length protein reaches the cell surface. Should functional testing confirm near-complete exon skipping with negligible residual protein, splice-modulating antisense oligonucleotides designed to promote exon 13 inclusion represent a potential investigational strategy that would require preclinical validation. Since 2020, the FDA has expanded the ETI label to include additional *CFTR* mutations based on *in vitro* responsiveness data (Clancy et al., 2019); however, nearly all variants tested were characterized in populations of European descent, and systematic theratyping of under-characterized CFTR variants reported in East Asian populations remains an unmet need.

In populations where early detection and CFTR modulators are available, predicted median survival for patients homozygous for F508del now exceeds 70 years (Lopez et al., 2023). Both genetically confirmed siblings died of respiratory failure in adolescence. Whether modulator therapy could have benefited them remains unknown and requires consideration of three distinct barriers. First, the 15-year diagnostic delay precluded access to specialized CF care including nutritional optimization, airway clearance, aggressive antimicrobial therapy, longitudinal monitoring, and timely transplant evaluation—interventions that improve survival independent of modulator therapy. Second, c.579 + 1_579+2insACAT is predicted to be a null allele; in the absence of a sufficiently expressed, membrane-localized, and pharmacologically responsive CFTR protein product from this allele, currently approved CFTR modulators would not be expected to provide clinical benefit. Third, even if genotype-directed therapy were indicated, CFTR modulators remain unavailable in mainland China, representing a systemic access barrier affecting all Chinese CF patients regardless of eligibility. Globally, an estimated 105,000 individuals are living with diagnosed CF across 94 countries, yet modulator therapy remains commercially unavailable in much of Asia, Africa, and Latin America (Guo et al., 2024). Moreover, modulator use requires a confirmed genetic diagnosis, meaning that undiagnosed patients cannot benefit.

Several measures could help break this cycle. Most immediately, expansion and decentralization of sweat chloride testing capacity—with standardized quality control per CLSI/CF Foundation guidance and dedicated technician training—represents the lowest-cost, highest-yield intervention to reduce CF underdiagnosis, as it is faster and cheaper to scale than newborn-screening infrastructure and directly addresses the confirmatory-testing gap. In parallel, clinician education and awareness campaigns targeting primary care and general pediatrics, coupled with clearly defined referral pathways to CF centers, are essential to ensure that children with recurrent respiratory disease or unexplained bronchiectasis are referred for appropriate testing. Longer-term, incorporation of CF into newborn screening programs would shift the point of diagnosis to the presymptomatic period; however, IRT-only protocols have limited positive predictive value, and second-tier IRT/DNA algorithms rely on mutation panels designed predominantly for European-descent populations that would miss the majority of Chinese-specific CFTR variants (Shen et al., 2022). Population-adapted panels incorporating East Asian high-frequency variants would be a prerequisite for effective implementation in China, and feasibility in low-prevalence populations requires further evaluation. Additionally, establishment of regional collaborative networks for CFTR variant functional characterization, pooling patient-derived samples from East Asian centers, could generate the data needed for modulator eligibility determinations. Finally, international pricing agreements and voluntary licensing could expand CFTR modulator accessibility in low- and middle-income countries where these therapies remain commercially unavailable.

This report is limited by the absence of functional studies on the identified variants and the short interval between diagnosis and death, which precluded a trial of modulator therapy. Specifically, direct RNA or protein-level functional characterization of c.579 + 1_579+2insACAT has not been performed; its predicted null effect is based on the disruption of the invariant canonical splice donor dinucleotide and requires future transcript-level confirmation. Upper gastrointestinal endoscopy was not performed, precluding assessment of esophageal varices despite ultrasound evidence of portal hypertension. Additionally, fat-soluble vitamin levels (A, D, E, K) and CF-related diabetes screening were not performed, as clinical management prioritized acute respiratory stabilization and transplant evaluation given the patient’s critical condition. Nasal nitric oxide was measured using an electrochemical sensor-based device rather than a chemiluminescence analyzer, which has been more extensively validated for this measurement. Nonetheless, this family demonstrates how diagnostic delay, insufficient variant characterization, and treatment inaccessibility may collectively contribute to poor outcomes in CF patients carrying rare, uncharacterized variants: patients who are never diagnosed cannot be genotyped, those who are genotyped may carry variants without theratyping data, and even those with confirmed diagnoses cannot access modulator therapy. Expanding diagnostic networks, characterizing population-specific variants, and improving drug accessibility must be pursued in parallel to break this cycle.

## Data Availability

The raw data supporting the conclusions of this article will be made available by the authors, without undue reservation.
